# Clinical and diagnostic characteristics of the development of hepatocardial syndrome in black and white cows in the early lactation period

**DOI:** 10.14202/vetworld.2022.2259-2268

**Published:** 2022-09-23

**Authors:** Yury Vatnikov, Andrey Rudenko, Larisa Gnezdilova, Elena Sotnikova, Varvara Byakhova, Elena Piven, Evgeny Kulikov, Aleksandr Petrov, Stanislav Drukovskiy, Olesya Petrukhina

**Affiliations:** 1 Department of Veterinary Medicine, Agrarian and Technological Institute, Peoples’ Friendship University of Russia (RUDN University), Moscow, Russia; 2 Department of Veterinary Medicine, State University of Food Production, Moscow, Russia; 3 Department of Diseases, Diagnostics, Therapy, Obstetrics and Reproduction of Animals, Moscow State Academy of Veterinary Medicine and Biotechnology - MVA Named after K.I. Skryabin, Moscow, Russia; 4 Department of Public Health, Healthcare, and Hygiene, Institute of Medicine, Peoples’ Friendship University of Russia (RUDN University), Moscow, Russia

**Keywords:** early lactation, heart dysfunction, hepatocardial syndrome, ketosis in cows, liver pathology, transitional period

## Abstract

**Background and Aim:**

It is known that during the early postpartum and lactation periods in dairy cows, metabolic disorders develop, that is, ketosis, which can lead to secondary damage to internal organs. Therefore, it is important to address the issues of changing the lactating cows’ clinical, laboratory, and physiological parameters regarding the development of hepatocardial syndrome. This study aimed to provide clinical and diagnostic justification for developing hepatocardial syndrome in highly productive dairy cows.

**Materials and Methods:**

The study was conducted on 20 black and white cows in the early postpartum period (7–10 days after birth), with a milk production level of >4500 kg of milk during the previous lactation period, a positive result in the formol colloid sedimentary test, the presence of deafness and splitting of heart sounds, changes in the size, or increased pain sensitivity of the percussion field of the liver. Clinically healthy dairy cows in the early postpartum period were used as controls (n = 24). Clinical, electrocardiographic, echocardiographic, and biochemical parameters were also evaluated.

**Results:**

Dairy cows with hepatocardial syndrome developed arterial hypertension and sinus tachycardia, which led to a significant decrease in PQ and QT intervals at ECG. A significant increase in the diastolic size of the interventricular septum, systolic size of the free wall of the left ventricle, and diastolic and systolic sizes of the left ventricle and a significant decrease in the shortening fraction of the left ventricular myocardium were observed in the cows due to the development of hepatocardial syndrome. The affected cows demonstrated a significant increase in serum activity of gamma-glutamyl transferase, alanine aminotransferase, lactate dehydrogenase, creatine phosphokinase, alkaline phosphatase, troponin, malondialdehyde, diene conjugates, and ceruloplasmin and a decrease in glucose concentration. In addition, they demonstrated decreased activity of superoxide dismutase, catalase, glutathione peroxidase, and glutathione reductase.

**Conclusion:**

Hepatocardial syndrome in dairy cows occurs due to ketosis, characterized by arterial hypertension, sinus tachycardia, a moderate decrease in myocardial contractility, oxidative stress, and cytolysis of cardiomyocytes and hepatocytes. Therefore, the control and prevention of the development of hepatocardial syndrome will make it possible to maintain the productive health and longevity of dairy cows.

## Introduction

Breeding dairy cattle are important for developing the agricultural sector [1–3]. The current state of development in dairy farming is characterized by an increase in the productivity of cows, a decrease in feed costs per unit of production, and an improvement in its quality [4–6]. In recent years, the genetic potential of dairy cows has improved significantly [7, 8]. The result is the creation of highly productive herds in many farms, where milk yield per cow is more than 6000 kg per lactation. A high metabolism level in highly productive cows cannot be maintained without the organization of complete nutritive rations, which provide the optimal supply of animals with energy, nutrients, and biologically active substances [9]. Simultaneously, a level of productivity close to the genetically programmed level is achieved, the health of animals is preserved, and the duration of their operation is increased. Any violation of the technology of keeping and feeding highly productive cows, especially in the period close to calving and in the 1^st^ weeks of the postpartum period, leads not only to a decrease in productivity but also to the development of ketosis, myocardial dystrophy, and hepatodystrophy [10–14]. In unproductive animal species, pathologies of the liver and heart are also extremely common and constitute a significant problem in veterinary medicine [15–19].

Chronic liver pathologies can negatively affect cardiovascular system function [20]. Changes in liver function play an important role in predicting the outcomes of severe chronic heart failure [21]. Cases of myocarditis and atherosclerosis have been described in chronic hepatitis [22]. Cases of cirrhotic cardiomyopathy and congestive hepatopathy in chronic heart failure have also been described [23]. Metabolic pathologies in highly productive cows are associated with damage to the liver and myocardium [24, 25]. Acute oleander poisoning in calves leads to simultaneous liver and myocardial injuries [26, 27]. Similar pathoanatomical changes have been described in poisoning with *Senna obtusifolia* [28] and fumonisins [29]. Hepatomegaly, increased activity of hepatic aminotransferases, and dilation of the hepatic veins, caudal vena cava, and portal vein are found in 88.1% of cows with traumatic reticulopericarditis [30, 31]. Liver and myocardial lesions were observed in calves with iron and zinc deficiency [32].

Studies on the pathogenesis and methods of correcting hepatocardial syndrome in animals have not been conducted. Therefore, the study of the pathogenetic mechanisms of the occurrence of hepatocardial syndrome and early informative methods for its diagnosis is relevant, as it will solve the issue of complex treatment and prevention of pathology of internal organs in highly productive dairy cows in the transitional and early lactation periods.

This study aimed to give a clinical and diagnostic justification for developing hepatocardial syndrome in highly productive dairy cows in the early lactation period.

## Materials and Methods

### Ethical approval

The study was conducted in accordance with the recommendations of the Declaration of Helsinki and approved by the Ethics Committee of the University (EA1/1811, October 05, 2020).

### Study period and location

The study was conducted from February 2021 to January 2022 at the Moscow region farms, Peoples’ Friendship University of Russia (RUDN University), Moscow State University of Food Production and MVA Named after K.I. Skryabin.

### Study design

The cross-sectional study was conducted on 223 black and white cows, including 20 cows with hepatocardial syndrome in one farm herd.

### Inclusion criteria

The following criteria were included in the study: Early postpartum period (7–10 days after birth), a milk production level of >4500 kg of milk in the previous lactation, a positive formol colloid sediment test, deafness, and splitting of heart sounds, a change in size, or increased pain sensitivity of the percussion field of the liver. The control group comprised clinically healthy animals (n = 24).

### Exclusion criteria

The following criteria were excluded from the study: Acute inflammatory processes (mastitis, endometritis, traumatic pericarditis, laminitis, reticuloperitonitis, pleurisy, and pneumonia), infectious and parasitic diseases, and incomplete clinical, laboratory, and instrumental data.

### Sample size determination

It is necessary to calculate the minimum sample size to study the pathogenesis and clinical, laboratory, and physiological diagnostic criteria. When analyzing the literature and evaluating indicators in highly productive cows, it was found that the ratio of the clinically significant difference in group mean values to the standard deviation should be at least 0.9 [33]. Then, at a statistical significance level of 0.05 and a study power of 0.80, the minimum sample size should be at least 20 in both the experimental and control groups.

### Clinical methods

It includes thermometry, palpation, percussion, physical examination, tonometry, electrocardiography (ECG), echocardiography, and biochemical analysis of the blood sera. In addition, fatness was assessed visually and by palpation of the vertebral column in the lumbar region, sacrum, and the first caudal vertebrae. A 5-point system was used, with a step of ±0.25 points, to assess the degree of fatness in dairy cows [34].

### Tonometry

Blood arterial pressure was measured using high-resolution oscillometry of the tail artery. The cuff size corresponded to 40.0% of the tail circumference. Blood pressure measurements were performed 5–7 times consecutively, except for abnormal results with a deviation of more than 10.0%, and the arithmetic mean value was calculated [35]. A pet mean arterial pressure (MAP) graphic II veterinary tonometer (Cardio Command Inc., USA) was used.

### Electrocardiography

Electrocardiograms of clinically healthy cows and cows with hepatocardial syndrome were recorded directly on the farm from 9.00 to 12.00 am. The study was conducted during the period of the complete rest of the animals, and the impact of stress factors was minimized. The animal’s skin was first degreased with 95° ethyl alcohol to apply the electrodes, and conductive gels were then applied to its surface to optimize the electrical contact and obtain good-quality electrocardiograms. A standard electrocardiogram was obtained using precordial leads [36]. The positive electrode was located at the level of the 5^th^ left intercostal space caudal to the olecranon; the negative electrode was located in the lower 1/3 of the left part of the neck in the projection of the jugular groove; the third electrode was located at the level of the left humeroscapular joint. We used an EK1T-04 Midas ECG device (Russian Federation) (recording speed, 50 mm/s; gain, 1 mV/10 mm).

### Echocardiography

Echocardiograms were recorded on the farm from 9.00 to 12.00 am without sedation, in a standing position with the right thoracic limb abducted forward [37]. In this study, the right-sided parasternal projections were used in the long and short axes of the left ventricle. A conductive gel was applied to the skin surface. The transducer was located 5–10 cm above the right olecranon in an area of 3–4 intercostal spaces. To optimize the quality of the ultrasound image, we adjusted the depth, focal length, and signal amplification (overall and by the scanning depth). The images were obtained in the following projections: Right cranial long-axis parasternal projection to assess the outflow tract of the right ventricle; right caudal long-axis parasternal projection to assess the left chambers of the heart and the aortic root (Ao); right parasternal projection along the short axis at the levels of the aortic valve (assessment of the size of the aorta and left atrium); and tendon chords of the mitral valve (assessment of the size of the interventricular septum, parietal wall, and left ventricle). The following echocardiographic parameters were measured: Left atrial size, Ao diameter, interventricular septum in diastole (IVSd), interventricular septum in systole, left ventricular free wall in diastole, left ventricular free wall in systole (LVWs), left ventricular diameter in diastole (LVDd), left ventricular diameter in systole (LVDs), and left ventricular fraction shortening (FS). We used a Mindray M7 portable ultrasound scanner (Mindray, China) with a P4–2s sectoral phased probe (scanning frequency 1.3–4.7 MHz).

### Biochemical methods

Blood sampling was performed in the morning before the animals were fed. Blood samples were collected from the coccygeal vein into vacuum tubes using a blood coagulation activator. Using a semi-automatic biochemical analyzer Stat Fax 1904 Plus (Awareness Technology, USA), the activity of alanine aminotransferase (ALT) and aspartate aminotransferase (AST) was determined in blood serum using the Reitman-Frankel method, gamma-glutamyl transpeptidase – by reaction with α-γ-glutamyl-4-nitroaniline (Szasz method), lactate dehydrogenase (LDH) – by kinetic method, creatine phosphokinase (CPK) – by enzymatic method, alkaline phosphatase (ALP), urea – by color reaction with diacetyl monoxime, creatinine – by Yaffe reaction according to Popper’s method, cholesterol – by the enzymatic colorimetric method, triglycerides – by the turbidimetric method, total protein – by the biuret reaction, and albumins – by the nephelometric method using standard biochemical kits (Olvex Diagnosticum, Russia). The concentration of glucose and ketone bodies in the blood was measured using a FreeStyle Optium Xceed glucometer (Abbott Diabetes Care, China). The cardiac troponin concentration in the blood serum was determined by chemiluminescent immunoassay on microparticles using an Architect i2000 analyzer (Abbott, USA). The concentration of malondialdehyde in the blood serum was determined using thiobarbituric acid and ceruloplasmin in reaction with phenylenediamine dihydrochloride according to the following method [38]. The serum activities of superoxide dismutase, catalase, glutathione reductase, and glutathione peroxidase were measured according to standard procedures [39]. Diene conjugates in blood serum were determined using a method based on determining the content of lipid peroxidation products by absorption of monochromatic light flux in the ultraviolet region of the spectrum using a lipid extract at a wavelength of 220 nm [40]. A UNICO-2802S spectrophotometer (United Products and Instruments, Inc., USA) was used.

### Statistical analysis

During the primary statistical processing, the normality of the distribution of the obtained digital data was preliminarily assessed using the Shapiro–Wilk test. When comparing the two groups whose numerical indicators did not correspond to the normal distribution of signs, the non-parametric Mann–Whitney U-test was used. Furthermore, 95% confidence intervals (CIs) were calculated. The difference between the indicators of the cows in the experimental and control groups was considered statistically significant at p < 0.05. All calculations were performed on a personal computer using the statistical program STATISTICA 7.0 (StatSoft, USA).

## Results

Clinical examination of 223 heads of black and white breed cows during early lactation revealed 20 heads with symptoms of damage to the cardiovascular system and liver, which amounted to 8.7%. To determine the main causes of morbidity, we analyzed the milk productivity of cows and other important clinical parameters (Table-1). In clinically healthy cows, for the previous lactation, milk yield averaged 5103 ± 1542 kg (95% CI 4452–5754 kg), while it was 7702 ± 2516 kg (95% CI 6524–8880 kg) in cows with hepatocardial syndrome, which was significantly higher. Moreover, in 75.0% of sick cows, milk yield exceeded 6000 kg. Furthermore, 90% of cows with hepatocardial syndrome were in operation for more than 3 lactations.

**Table-1:** Clinical indicators for hepatocardial syndrome in dairy cows.

Indicator	Control (n = 24)		Hepatocardial syndrome (n = 20)	
	
	M ± SD	95% CI	M ± SD	95% CI
Milk yield for the previous lactation, kg	5103 ± 1522	4552–5754	7702 ± 2516***	6524–8880
Average daily milk yield, kg	16.8 ± 5.1	14.6–19.0	25.3 ± 8.2***	21.4–29.1
Temperature, °C	38.4 ± 0.3	38.3–38.5	38.2 ± 0.2	38.1–38.3
Pulse, min-1	72.3 ± 5.7	69.9–74.7	92.8 ± 10.5***	87.9–97.7
Respiration, min-1	15.3 ± 2.1	14.4–16.2	21.7 ± 3.8***	19.9–23.5
Rumination, 5 min-1	6.2 ± 1.2	5.6–6.7	2.9 ± 0.9***	2.4–3.3
SAP, mmHg	116.5 ± 9.0	112.8–120.3	140.3 ± 10.8***	135.2–145.4
DAP, mmHg	54.4 ± 12.8	49.0–59.8	78.9 ± 7.0***	75.6–82.1
AAP, mmHg	75.1 ± 11.0	70.5–102.5	97.8 ± 10.2***	92.9–102.5

*(p < 0.05), **(p < 0.01), and

*** (p < 0.001): Indicate the significant difference between the parameters of the group of animals with hepatocardial syndrome and clinically healthy animals (Mann–Whitney U-test). M=Mean, SD=Standard deviation, CI=Confidence interval, SAP=Systolic arterial pressure, DAP=Diastolic arterial pressure, AAP=Average arterial pressure

The system for keeping cattle on the farm is a year-round stall (without walking). The average daily productivity in clinically healthy dairy cows was 16.8 ± 5.1 kg of milk (95% CI 14.6–19.0). In dairy cows with hepatocardial syndrome, the average daily milk yield was significantly higher and amounted to 25.3 ± 8.2 kg (95% CI 21.4–29.1). In the cows’ diets, a reduced ratio of sugar: protein (0.29–0.42:1) and sugar + starch: protein (1.1–1.22:1) was observed.

In cows with hepatocardial syndrome (Table-1), body condition was average or above average (body condition score was 4.4 ± 0.3). No edema was observed in the dewlap, submandibular space, or distal extremities. On palpation, the heartbeat was rhythmic, localized, and weakened. The weakening of tones was detected in 95.0% and splitting in 30.0% of cows using auscultation. In cows with signs of hepatocardial syndrome, 92.8 ± 10.5 heart contractions per min (95% CI 87.9–97.7 r/min) were observed, which was significantly higher (p < 0.001) than that in clinically healthy cows. Tachycardia (>100 beats/min) was observed in 30.0% of sick animals. In addition, cows with hepatocardial syndrome had significantly increased respiratory rate (RR) (1.42 times; p < 0.001), systolic (1.2 times; p < 0.001), diastolic (1.45 times; p < 0.001), and mean arterial blood pressure (1.3 times; p < 0.001) and significantly decreased frequency of rumen contractions (2.14 times; p < 0.001) compared with clinically healthy cows.

Table-2 shows the results of the electrocardiographic studies in cows that developed hepatocardial syndrome.

**Table-2:** Electrocardiographic parameters in cows with hepatocardial syndrome.

ECG Intervals	Control (n = 24)		Hepatocardial syndrome (n = 20)	
	
	M ± SD	95% CI	M ± SD	95% CI
P,ms	76.8 ± 6.4	74.1–79.5	75.5 ± 6.5	72.4–78.5
PQ,ms	163.0 ± 20.5	154.4–171.7	138.5 ± 14.4***	131.7–145.3
QRS,ms	69.3 ± 8.0	65.9–72.7	69.2 ± 6.0	66.4–72.0
QT,_MS_	344.3 ± 34.6	288.9–327.4	308.2 ± 41.1***	288.9–327.4
PII,_M_V	0.20 ± 0.07	0.17–0.23	0.15 ± 0.05*	0.12–0.17
SII,_M_V	−0.59 ± 0.18	−0.67–(−0.52)	−0.4 ± 0.25*	−0.51–(−0.28)
TII,_M_V	0.30 ± 0.15	0.23–0.37	0.20 ± 0.11*	0.15–0.26
ST,_M_V	0.01 ± 0.06	−0.03–0.02	−0.03 ± 0.08	−0.07–0.01

* (p < 0.05), **(p < 0.01), and

*** (p < 0.001): Indicate the significant difference between the parameters of the group of animals with hepatocardial syndrome and clinically healthy animals (Mann–Whitney U-test). M=Mean, SD=Standard deviation, CI=Confidence interval

All control cows had a regular sinus rhythm with a normal heart rate (sinus normocarbia). Sinus rhythm was diagnosed in all cows that developed hepatocardial syndrome. However, it was found that there was a significant shortening of the PQ interval (by 1.18 times; p < 0.001) and QT (by 1.12 times; p < 0.001) intervals and a decrease in p wave voltage (by 1.33 times; p < 0.001), S (by 1.48 times; p < 0.001), and T (by 1.5 times; p < 0.001) on the electrocardiograms of cows with hepatocardial syndrome compared with clinically healthy cows. We did not find clinically significant changes in excitability, conduction, automatism, or myocardial repolarization in animals from different experimental groups.

**Figure-1:**
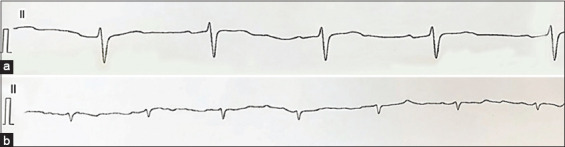
(a) Electrocardiographic parameters of healthy, highly productive cows (b) with the development of hepatocardial syndrome. Velocity 50 mm/s, voltage 1 cm/1 mV.

The nature of the changes in electrocardiographic parameters in cows with hepatocardial syndrome is shown in Figure-1. Cows with hepatocardial syndrome, non-specific changes occurred on electrocardiograms, which are manifested by a decrease in the voltage of the teeth and a tendency to accelerate the heart rate (Figure-1).

The results of changes in basic echocardiographic parameters in cows with hepatocardial syndrome are shown in Table-3. Cows with hepatocardial syndrome, compared with clinically healthy ones, a significant increase in IVSd (by 1.11 times; p < 0.05), LVWs (by 1.1 times; p < 0.05), LVDd (by 1, 1 times; p < 0.01), and LVDs (1.2 times; p < 0.05) and a significant decrease in FS (1.3 times; p < 0.001) were observed. However, these changes were not accompanied by clinically significant remodeling of the heart chambers or hemodynamic disturbances and did not lead to the development of congestive heart failure syndrome.

**Table-3:** Echocardiographic parameters in cows with hepatocardial syndrome.

Indicator	Control (n = 24)		Hepatocardial syndrome (n = 20)	
	
	M ± SD	95% CI	M ± SD	95% CI
LA cm	7.6 ± 0.7	7.3–7.8	7.7 ± 0.8	7.3 8.0
Ao,cm	6.0 ± 0.3	5.9–6.2	6.0 ± 0.3	5.8–6.2
LAAo, units	1.2 ± 0.1	1.2–1.3	1.3 ± 0.2	1.2–1.4
IVSd,cm	1.8 ± 0.2	1.7–1.9	2.0 ± 0.2*	1.9–2.1
IVSs,cm	2.8 ± 0.2	2.7–2.9	2.9 ± 0.2	2.8–3.0
LVWdcm	1.9 ± 0.2	1.9–2.0	2.0 ± 0.2	1.9–2.1
LVWs,cm	2.9 ± 0.2	2.7–3.0	3.1 ± 0.2*	3.0–3.2
LVDd,cm	7.7 ± 0.6	7.5–8.0	8.4 ± 0.6**	8.1–8.7
LVDs,cm	5.0 ± 0.5	4.7–5.2	6.1 ± 0.8**	5.7–6.4
FS, %	36.1 ± 6.0	33.5–38.6	28.2 ± 7.4***	24.7–31.7

* (p < 0.05),

** (p < 0.01), and

*** (p < 0.001): Indicate the significant difference between the parameters of the group of animals with hepatocardial syndrome and clinically healthy animals (Mann–Whitney U-test). M=Mean, SD=Standard deviation, CI=Confidence interval, LA=Left atrium, Ao=Aortic root, IVSd=Interventricular septum in diastole, IVSs=Interventricular septum in systole, LVWs=Left ventricular free wall in systole, LVDd=Left ventricular diameter in diastole, LVWd=Left ventricular free wall in diastole, LVDs=Left ventricular diameter in systole, FS=Fraction shortening

The results of biochemical parameters of blood serum in cows with hepatocardial syndrome are shown in Table-4.

**Table-4:** Biochemical parameters of blood serum in cows with hepatocardial syndrome.

Indicator	Control (n = 24)		Hepatocardial syndrome (n = 20)	
	
	M ± SD	95% CI	M ± SD	95% CI
GGTP, U/l	21.3 ± 3.7	19.7–22.8	27.9 ± 5.5***	25.3–30.4
ALT, U/l	52.5 ± 20.6	43.8–61.2	197.0 ± 73.5***	162.6–231.4
AST, U/l	75.3 ± 17.4	67.9–82.7	140.4 ± 36.5***	123.3–157.4
LDH, U/l	258.3 ± 84.7	222.6–294.1	523.5 ± 184.1***	437.3–609.6
CPK, U/l	92.8 ± 26.9	81.4–104.1	215.4 ± 84.7***	175.7–254–9
ALP, U/l	132.8 ± 45.4	113.6–152.0	223.8 ± 94.1***	179.7–267.8
Troponin, ng/mL	0.04 ± 0.05	0.02–0.06	0.16 ± 0.05***	0.13–0.19
Urea, mmol/l	4.0 ± 1.1	3.5–4.4	3.9 ± 1.3	3.2–4.5
Creatinine, mmol/l	116.2 ± 30.8	103.2–129.2	134.5 ± 37.5	116.9–152.0
Glucose, mmol/l	3.3 ± 0.9	2.9–3.7	2.2 ± 0.6***	1.9–2.5
Ketone bodies, mmol/l	0.7 ± 0.2	0.6–0.8	2.1 ± 0.7***	1.8–2.4
Cholesterol, mmol/l	3.2 ± 0.7	2.9–3.5	2.1 ± 0.6**	1.8–2.3
Triglycerides, mmol/l	0.8 ± 0.3	0.6–0.9	1.1 ± 0.3*	0.9–1.2
Total protein, g/l	74.4 ± 4.4	72.6–76.3	80.3 ± 6.2**	77.4–83.2
Albumins, g/l	31.9 ± 2.2	30.9–32.8	29.9 ± 2.8*	28.5–31.2
Malondialdehyde, mmol/l	1.4 ± 0.5	1.2–1.7	2.9 ± 0.5***	2.7–3.2
Ceruloplasmin, mmol/l	1.6 ± 0.4	1.4–1.8	3.0 ± 0.4***	2.8–3.2
Superoxide dismutase, U/mL	77.3 ± 20.6	68.6–85.9	40.4 ± 15.1***	33.3–47.5
Catalase, U/mL	0.9 ± 0.3	0.7–1.1	0.4 ± 0.3***	0.3–0.6
Glutathione reductase, U/mL	0.9 ± 0.2	0.8–1.0	0.7 ± 0.2*	0.6–0.8
Glutathione peroxidase, U/mL	3.3 ± 0.7	2.9–3.6	2.5 ± 0.7**	2.2–2.8
Diene conjugates, arb. units/mL	2.0 ± 0.3	1.9–2.1	3.4 ± 0.8***	3.0–3.8

* (p < 0.05),

** (p < 0.01), and

*** (p < 0.001): Indicate the significant difference between the parameters of the group of animals with hepatocardial syndrome and clinically healthy animals (Mann–Whitney U-test). M=Mean, SD=Standard deviation, GGTP=Gamma-glutamyl transpeptidase, ALT=Alanine aminotransferase, AST=Aspartate aminotransferase, LDH=Lactate dehydrogenase, CPK=Creatine phosphokinase, ALP=Alkaline phosphatase

Black and white breed cows with hepatocardial syndrome, compared with clinically healthy animals, showed a significant increase in serum activity of GGT (1.31 times; p < 0.001), ALT (7.75 times; p < 0.001), AST (1.86 times; p < 0.001), LDH (2.03 times; p < 0.001), CPK (2.33 times; p < 0.001), ALP (by 1.69 times; p < 0.001), troponin concentrations (by 4.0 times; p < 0.001), ketone bodies (by 3.0 times; p < 0.001), triglycerides (by 1, 38 times; p < 0.05), total protein (1.1 times; p < 0.01), and diene conjugates (1.7 times; p < 0.001). Simultaneously, in sick cows, the concentration of glucose in the blood serum (by 1.5 times; p < 0.001), cholesterol (by 1.5 times; p < 0.01), and albumin (by 1.1 times; p < 0.05) decreased, and a significant decrease in the serum activity of superoxide dismutase (1.91 times; p < 0.001), catalase (2.25 times; p < 0.001), glutathione reductase (1.29 times; p < 0.05), and glutathione peroxidase (1.32 times; p < 0.01) was observed (Table-4).

**Figure-2:**
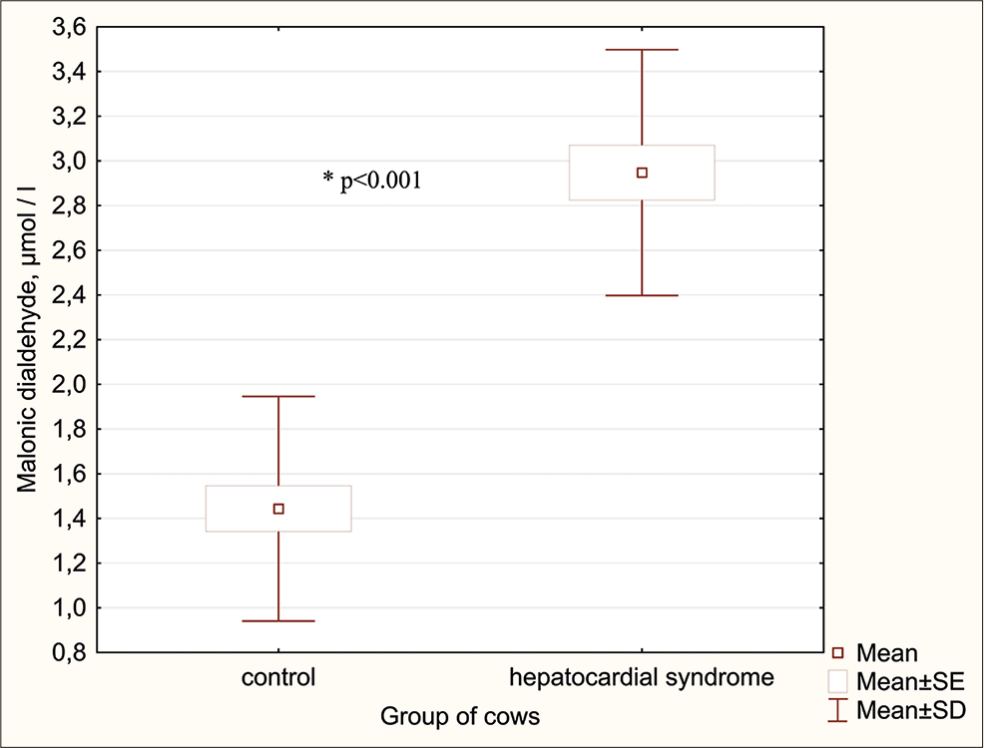
Serum concentration of malondialdehyde and ceruloplasmin in cows with hepatocardial syndrome.

The results of the concentrations of malondialdehyde and ceruloplasmin in cows with hepatocardial syndrome are shown in Figure-2. Black and white breed cows with hepatocardial syndrome, compared with clinically healthy animals, a significant increase in the concentration of malondialdehyde (2.07 times; p < 0.001) and ceruloplasmin (1.9 times; p < 0.001) was found.

## Discussion

Numerous studies have been conducted on various internal organ and metabolic diseases in highly productive cows [1, 2, 7, 41]. However, these studies used a monocausal approach: One pathology to one method of treatment and prevention. In veterinary practice, one often has to deal with cases of developing multiple pathologies of internal organs. There is practically no data on the theoretical and clinical justification for the causes of their development, pathogenesis, diagnostic criteria, and methods of treatment and prevention. The study of the combined damage to the liver and myocardium (hepatocardial syndrome) that occurs in highly productive dairy cows due to metabolic diseases in the transitional and early lactation periods is of particular interest. In veterinary medicine, hepatocardial syndrome has not been described yet. There have been reports on developing simultaneous damage to the heart and liver in cases of pericarditis, infections, invasion, metabolic disorders, endocrinopathies, and toxicosis [26, 27, 30, 31]. Therefore, a real scientific problem requires a timely solution. The course and mechanisms of the development of hepatocardial syndrome in animals can be used as a model of a similar pathological process in humans.

Our study showed that hepatocardial syndrome in dairy cows occurred at a frequency of 8.7%. High milk productivity is a risk factor for developing hepatocardial complications in cows. Physical inactivity aggravates metabolic disorders in sick animals. We found that an imbalance in cows’ diets with high milk productivity reduces the absorption of energy and plastic material, which leads to the development of ketosis. The literature also shows that this pathology can be complicated by metabolic acidosis and osteodystrophy [42, 43]. Hypoglycemia and increased concentration of ketone bodies were observed in the blood of cows with hepatocardial syndrome. Therefore, in our study, cows were diagnosed with a clinical form of ketosis, which was the main cause of hepatocardial complications.

Metabolic disorders in the affected cows trigger free radical lipid peroxidation, which is the main energy source for the heart muscle. Our study showed that in the blood serum of cows with hepatocardial syndrome, the concentrations of malondialdehyde and diene conjugates significantly increased, indicating an increase in lipid peroxidation processes. In contrast, the serum activity of superoxide dismutase, catalase, glutathione reductase, and glutathione peroxidase significantly decreased, indicating system inhibition of the antioxidant protection of the body. Oxidative stress is an important pathogenic mechanism in the body of dairy cows with ketosis, which has also been demonstrated in other studies [44, 45].

In addition, a systemic inflammatory reaction can develop in cows with clinical and subclinical ketosis, which can induce secondary damage to internal organs [46–48]. In this case, the neurohumoral systems of the body are activated, and an excess amount of catecholamines is formed, which leads to a decrease in the glycogen reserve in cardiomyocytes, leading to the development of intracellular acidosis and a slowdown of energy conversion processes. Therefore, the myocardium does not receive the required amount of adenosine triphosphate and cannot fully perform its functions.

In this study, there was a significant increase in heart rate, systolic, diastolic, and MAP and a decrease in the time of atrioventricular conduction and electrical systole in cows with hepatocardial syndrome, which may indirectly indicate the activation of the sympathetic nervous system, adrenal, and renin-angiotensin-aldosterone system.

Violation of the functional state of the cardiovascular system in highly productive cows is caused by the accumulation of toxic products during the development of hepatodystrophy. Toxins in blood flow enter the coronary arteries that provide the heart muscle with nutrients and penetrate cardiomyocytes, disrupting their energy and metabolism. As a result of changes in the biochemical state of cardiomyocytes, the enzymes responsible for energy metabolism in cells (LDH, creatine kinase, and AST) and the constituent parts of cardiomyofibrils (cardiotroponins) are actively released into the intercellular space and then into the blood. Our study showed that in cows with hepatocardial syndrome, the activity of asparagine aminotransaminase, LDH, and CPK and cardiac troponin concentration significantly increased in the blood serum, indicating cardiomyocyte cytolysis syndrome. Liver damage in ketotic cows has also been reported [33, 42, 44, 46].

In this study, congestive heart failure syndrome development in cows with ketosis was not established. However, another study described 13 cases of acute congestive heart failure in cows in the early postpartum period with fatal outcomes [49]. In addition, human medicine has described peripartum cardiomyopathy in detail [22, 50–52]. The presence of a long-term metabolic disorder in the body of sick animals can deplete the compensatory and adaptive capabilities of the cardiovascular system and cause severe and irreversible changes in the myocardium.

Damaged myocardial cells cannot effectively conduct impulses from the sinus node to the ventricles, and some sections of the myocardium cannot conduct an impulse. In our study, electrocardiograms of cows with hepatocardial syndrome showed a decrease in T waves, indicating a change in ventricular repolarization processes. The electrocardiogram of cows with hepatocardial syndrome is characterized by a decrease in the duration of the cardiac cycle (RR interval) due to a decrease in the diastolic period (TP interval); a decrease in the time of atrioventricular conduction and the duration of the electrical ventricular systole; and a decrease in the voltage of the P, S, and T waves.

Because of tachycardia, the myocardium’s cardiac cycle and rest time are reduced. The myocardium accumulates its own incompletely oxidized toxic metabolic products, which increases the pathological effect. In the future, signs of increasing cardiac dysfunction can be diagnosed using clinical research methods. In sick cows, a weakening of the heart impulse and changes in heart sounds were detected, as shown in our studies.

Violation of the ratio between easily fermented carbohydrates and protein in the diets of highly productive cows during early lactation causes changes in the pH of the rumen content, which is characterized by increased synthesis of biologically active and toxic substances [46–48]. Increased concentrations of metabolic products and endogenous toxins can lead to hepatocyte dystrophy. The detoxification function of the liver decreases, and toxic substances accumulate in hepatocytes, leading to liver dystrophy. Incompletely oxidized toxic substances in blood flow enter myocardial cells, thereby disrupting intracellular metabolism in cardiomyocytes.

Therefore, a vicious pathogenic cycle is formed. Liver dysfunction causes myocardial dysfunction, and conversely, myocardial damage leads to the aggravation of liver failure. The literature describes cases in which chronic liver pathologies can negatively affect the cardiovascular system [50]. In contrast, changes in the functional state of the liver can play an important role in predicting the outcome of disease in severe heart failure syndrome [51]. In chronic hepatitis, there are frequent cases of inflammation of the heart muscles [22], and with the development of hepatodystrophy, dystrophic changes occur in the myocardium [52]. The presence of heart failure in chronic hepatopathy significantly worsens the prognosis [53]. In addition, chronic hepatopathy can initiate atherosclerosis and vessel wall remodeling [23]. Cirrhotic cardiomyopathy has also been described in humans [50, 54]. Systolic and diastolic dysfunction of the heart often develops with liver cirrhosis and the syndrome of QT interval prolongation on electrocardiograms [55].

A significant limitation of this study is the lack of histological examination results for myocardial and liver tissues in cows with hepatocardial syndrome. However, in our case, changes in the cardiovascular system and liver in highly productive cows in the transitional and early lactation periods were more functional. Therefore, culling or forced slaughter of animals was not required. In our subsequent work, we plan to study the nature of morphological changes in the liver and myocardial tissues of cows with hepatocardial syndrome.

In addition, ultrasound examinations can easily establish signs of hepatocardial syndrome in pets and other data related to the heart, liver, kidneys, and other internal organs. Furthermore, in future studies, we plan to study in detail the ultrasonographic characteristics of changes in the liver and other internal organs in highly productive cows with hepatocardial syndrome.

Prospects for further research on the development of multimorbid changes in the liver and myocardium include a detailed study of the pathogenesis of the development, formation, and progression of hepatocardial syndrome in an experimental model of laboratory animals.

## Conclusion

In the black and white breed dairy cows, hepatocardial syndrome may develop during the transitional and early lactation periods due to the development of clinical ketosis. In this study, hepatocardial complications were formed due to clinical ketosis, manifested by a significant increase in pulse rate, RR, and systolic, diastolic, and MAP. In contrast, there were no clinically significant changes in electrocardiographic parameters. Sinus tachycardia developed in dairy cows with hepatocardial syndrome, which led to a significant decrease in the time of atrioventricular conduction and the duration of electrical systole of the heart (PQ and QT intervals). The P, S, and T wave voltages also decreased significantly. In cows with hepatocardial syndrome, a significant increase in the diastolic size of the interventricular septum, systolic size of the free wall of the left ventricle, and diastolic and systolic sizes of the left ventricle and a significant decrease in myocardial contractility were observed. In addition, a significant increase in serum activity of GGT, ALT, LDH, CPK, and ALP, the concentration of ketone bodies, troponin, triglycerides, malondialdehyde, diene conjugates, and ceruloplasmin, a decrease in cholesterol concentration, albumin, and glucose, and decreased activity of superoxide dismutase, catalase, glutathione peroxidase, and glutathione reductase were observed. The control and prevention of the development of hepatocardial syndrome will make it possible to maintain dairy cows’ productive health and longevity.

## Authors’ Contributions

YV, AR, and EK: Conceptualized and designed the study. LG, ES, EP, and AP: Collected the samples. EK, VB, SD, OP, AR, and YV: Data analysis. YV, VB, EK, LG, and AR: Drafted the manuscript. All the authors have read and approved the final manuscript.
